# A multicenter retrospective evaluation of Chronic Myeloid Leukemia (CML) therapy in Austria assessing the impact of early treatment response on patient outcomes in a real-life setting: R-EFECT study

**DOI:** 10.1007/s00508-020-01690-1

**Published:** 2020-06-12

**Authors:** Andreas L. Petzer, Wolfgang R. Sperr, Veronika Buxhofer-Ausch, Thamer Sliwa, Stefan Schmidt, Richard Greil, Albert Wölfler, Petra Pichler, Clemens Dormann, Sonja Burgstaller, Christoph Tinchon, Alois Lang, Florian Goebel, Shanow Uthman, Niklas Muenchmeier, Peter Valent

**Affiliations:** 1 Internal Medicine I, Department of Hematology, with Stem Cell Transplantation, Hemostaseology and Medical Oncology, Ordensklinikum Linz Barmherzige Schwestern/Elisabethinen, Linz, Austria; 2 Department of Internal Medicine I, Division of Hematology & Hemostaseology and Ludwig Boltzmann Institute for Hematology and Oncology, Medical University of Vienna, Vienna, Austria; 3 Internal Medicine I Department of Hematology with Stem Cell Transplantation, Hemostaseology and Medical Oncology, Ordensklinikum Linz Elisabethinen, Linz, Austria; 4 Department of Medicine III: Hematology Oncology, Hanusch Hospital Vienna, Vienna, Austria; 5 Internal Medicine V: Hematology-Oncology, University of Innsbruck, Innsbruck, Austria; 6 Salzburg Cancer Research-Institute-CCCIT, Cancer Cluster Salzburg, Paracelsus Medical University Salzburg, Salzburg, Austria; 7 Division of Hematology, Department of Internal Medicine, Medical University of Graz, Graz, Austria; 8 Klinische Abteilung für Innere Medizin I, Universitätsklinikum St. Pölten—Lilienfeld, Karl Landsteiner Privatuniversität für Gesundheitswissenschaften, Dunant-Platz 1, St. Pölten, Austria; 9 Internal Medicine I, Department of Medical Oncology and Hematology, Ordensklinikum Linz Barmherzige Schwestern, Linz, Austria; 10 Abteilung für Innere Medizin IV, Hämatologie und Onkologie, Klinikum Wels-Grieskirchen, Wels, Austria; 11 Department of Hemato-Oncology, LKH Hochsteiermark-Leoben, Leoben, Austria; 12 Innere Medizin, Rotkreuzklinik Lindenberg, Lindenberg, Austria; 13 Oncology, Novartis Pharma GmbH, Vienna, Austria

**Keywords:** Chronic myeloiud leukemia, Chronic phase, Retrospective evaluation, Tyrosine kinase inhibitors, Clinical routine

## Abstract

**Background:**

Several clinical trials in chronic phase (CP) chronic myeloid leukemia (CML) showed that early response to tyrosine kinase inhibitor (TKI) treatment results in an improved long-term survival and progression-free survival. This study assessed whether patients achieving early treatment response (ETR; partial cytogenetic response or BCR-ABL1 mRNA ≤10% at 3 months) in daily practice also have a long-term survival benefit.

**Methods:**

The *R*etrospective *E*valuation o*f E*arly response in *C*ML for long-term *T*reatment outcome (R-EFECT), a multicenter, retrospective chart review, documented patients with newly diagnosed CML-CP starting first-line TKI therapy in routine clinical practice. The primary aim was to assess the 5‑year overall survival rate.

**Results:**

Of the 211 patients from 12 centers across Austria (January 2004–May 2010), 176 (median age, 56 years) were included in the analysis. All patients received first-line therapy with imatinib. Overall, 136 patients (77.3%) achieved ETR (ETR+ group), whereas 40 (22.7%) did not reach ETR (ETR− group). The ETR+ group had higher 5‑year overall survival (92.5% vs. 77.5%, *P* = 0.018) and progression-free survival (95.6% vs. 87.5%, *P* = 0.06) rates compared with the ETR− group. As expected, more patients in the ETR− group were switched to another TKI. At the last contact, 120 patients were still on imatinib and 44 had switched to another TKI (25 to nilotinib, 15 to dasatinib, and 4 to bosutinib).

**Conclusion:**

The data are in line with randomized trials demonstrating that ETR is associated with improved survival and thus confirmed these results in patients treated in daily clinical routine.

## Introduction

According to the European Leukemia Network (ELN) guidelines for chronic myeloid leukemia (CML), BCR-ABL1 transcript levels (addressed as BCR-ABL1 throughout) of ≤10% according to the international scale (IS, BCR-ABL1^IS^) in the peripheral blood or a partial cytogenetic response (pCyR), <35% Philadelphia (Ph)+ metaphases, are defined as optimal treatment response at 3 months [1]. At 6 months and 12 months, the BCR-ABL1^IS^ should be <1% and ≤0.1%, respectively, or alternatively a complete cytogenetic response (CCyR; 0% Ph+ metaphases) at ≥6 months should be achieved to be classified as optimal response [1].

Several clinical trials have shown that patients with CML in chronic phase (CP) who achieve early molecular response (EMR, BCR-ABL1^IS^ ≤10% at 3 months) with tyrosine kinase inhibitor (TKI) therapy have better long-term responses, overall survival (OS), and progression-free survival (PFS) [2–10]; however, these data were obtained in patients selected for clinical trials. Therefore, it was of particular interest whether the survival benefit observed in these studies translates to patients treated in daily clinical practice.

This article presents data from a multicenter, retrospective analysis that assessed whether the achievement of an early treatment response (ETR) results in an improved survival in patients receiving TKI in clinical routine.

## Patients and methods

### Study design and patients

The *R*etrospective *E*valuation o*f E*arly response in *C*ML for long-term *T*reatment Outcome (R-EFECT) study documented 211 patients with newly diagnosed CP-CML who received first-line TKI therapy in clinical routine between January 2004 and May 2010 in 12 centers across Austria. Only patients with documented BCR-ABL1 levels and/or % Ph+ metaphases at the 3‑month visit were assessed. Patients who had received short-term (for ≤3 months) cytoreduction (e.g. hydroxyurea) or those who were subsequently treated with second or third generation TKIs (when available) could also be included. Patients who participated in interventional clinical trials were excluded.

The aim was to document outcomes in the clinical practice. We assessed the OS rate at 5 years in patients who achieved ETR, compared with those who did not reach ETR. Additional aims were to define the OS rates in the ETR+ and ETR− groups, the rates of progression to accelerated phase/blast crisis (AP/BC) at 5 years, and treatment responses at 6 months. In addition, ETR and OS rates were assessed based on Sokal risk scores at baseline [11].

### Assessments and definitions

The ETR was defined as achievement of at least a pCyR (≤35% Ph+ metaphases) or BCR-ABL1 ≤10% or both at 3 months, which is in line with the optimal response defined in the 2013 ELN criteria [1]. Response categories at 6 months were defined as per 2013 ELN guidelines [1].

For evaluation of responses, priority was given to BCR-ABL1^IS^ values, which could be substituted by cytogenetic results if BCR-ABL1^IS^ values were unavailable or with raw (i.e. non-IS) BCR-ABL1 values if neither BCR-ABL1^IS^ nor cytogenetic data were available. This response evaluation was performed in this order, irrespective of whether additional parameters for evaluation were available and whether concordance between the response parameters were given or not. An exploratory analysis was performed to assess the concordance between BCR-ABL1^IS^ and BCR-ABL1^raw^ values [12].

A PFS was defined as the absence of progression to AP/BC (as per ELN guidelines) as labeled by the investigator [1] and OS and PFS rates were expressed as the percentage of all patients with data available for the respective endpoint.

### Statistical analysis

For all descriptive statistical comparisons of categorical data (frequencies and percentages) χ^2^-tests were used. Continuous data were compared using Mann-Whitney U-tests.

### Ethics

This study was designed, implemented and reported in accordance with the guidelines for good pharmacoepidemiology practices (GPP) of the International Society for Pharmacoepidemiology (ISPE 2008), the STROBE (strengthening the reporting of observational studies in epidemiology) guidelines [13], and with the ethical principles laid down in the Declaration of Helsinki. It was approved by the Ethikkommission des Landes Oberösterreich on 19 August 2015 (No. K‑79-15).

## Results

### Patient characteristics and initial therapy

Of the 211 patients, 176 (median age, 56 years; male, 59.7%, *n* = 105) were included in the analysis (Table 1). The remaining 35 patients were excluded due to lack of clearly documented BCR-ABL1 levels or cytogenetic data at the 3‑month visit.

**Table 1:** Baseline demographics and clinical characteristics

Parameter	*N* = 176
Age, years, median (range)	56 (18–90)
Male, *n* (%)	105 (59.7)
Sokal risk score, *n* (%)	
High	18 (10.2)
Medium	42 (23.9)
Low	50 (28.4)
N/A	66 (37.5)
Short-term initial CML treatment before start of imatinib, *n* (%)	
Hydroxyurea	41 (23.3)
Peginterferon alpha 2b	1 (0.6)
No previous treatment	90 (51.1)
N/A	44 (25)
Tyrosine kinase inhibitor treatment start (median, days after diagnosis)	8

*CML* chronic myeloid leukemia, *N/A* not assessed

Since imatinib was the only approved first-line TKI until the end of 2010, all patients assessed received first-line therapy with imatinib. Of the 176 patients, 164 (93.2%) started on 400mg/day, 11 patients (6.3%) started on lower doses, and 1 patient (0.6%) started on 600mg/day.

### Impact of ETR on OS and PFS

Overall, 136 patients (77.3%) achieved an ETR at 3 months of treatment (ETR+ group) and 40 patients (22.7%) did not (ETR− group). The BCR-ABL1^IS^ values were used for the analysis of ETR in 102 of 176 patients (58%), cytogenetic responses in 41 patients (23%) and BCR-ABL1^raw^ values in 33 patients (19%). Molecular and cytogenetic responses were available in 58 of 176 patients (33%). Of note, a high concordance of 86% (50/58 patients) was observed in these patients between molecular (i.e. BCR-ABL ≤10% versus >10%) and cytogenetic responses (≤35% Ph+ metaphases vs >35% Ph+ metaphases).

The median age was comparable in ETR+ and ETR− patients with 55 years and 59.5 years, respectively.

**Fig. 1:**
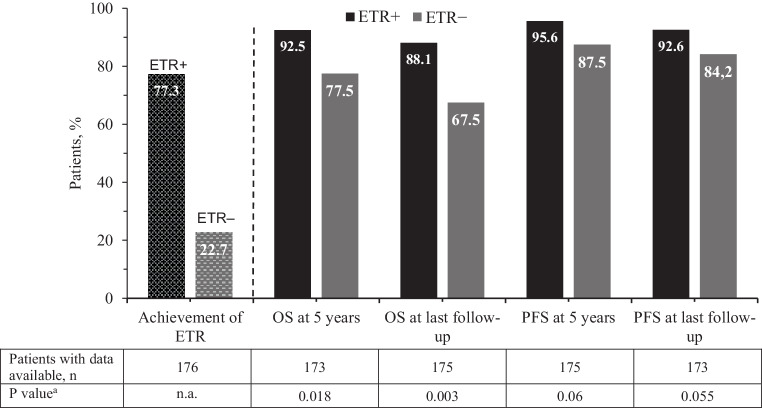
ETR status, OS and PFS at 5 years and at the last visit based on ETR status. *ETR* early treatment response (partial cytogenetic response and/or *BCR-ABL1* ≤ 10% at 3 months), *n.a.* not assessed, *OS* overall survival, *PFS* progression-free survival. ^a^For all descriptive statistical comparisons of categorical data (frequencies and percentages) chi square tests were used. Continuous data were compared using Mann-Whitney U-tests

The median duration until the last follow-up/visit was 94.5 months (7.9 years), which was similar between the ETR+ and ETR− subgroups (97.0 months and 91.5 months, respectively, censoring patients who had died within the documentation period in order to avoid bias through the higher number of deaths in the ETR− group). Patients in the ETR+ group had higher 5‑year OS and PFS rates compared with the ETR− group (OS: 92.5% vs. 77.5%, *P* = 0.018; PFS: 95.6% vs. 87.5%, *P* = 0.06). At the last follow-up, the differences between ETR+ and ETR− groups in OS and PFS were even more pronounced (OS: 88.1% vs. 67.5%, *P* = 0.003; PFS: 92.6% vs. 84.2%, *P* = 0.055) (Fig. 1).

At 5 years of follow-up, 19 deaths were reported, 10/136 (7.4%) in the ETR+ group and 9/40 (22.5%) in the ETR− group. In the ETR− group, 2 patients died within 12 months after diagnosis (early death). No early deaths occurred in the ETR+ group. Of the 19 deaths 4 (21%) recorded within 5 years were CML-related (3 in ETR+ group, 2.2%, 1 in the ETR− group, 2.5%). At the last follow-up, 29 deaths were reported, including 16 in the ETR+ group (11.8%) and 13 in the ETR−, group (32.5%). Of all the patients followed, 16 had progressed to AP/BC, including 10 in the ETR+ group (7.4%) and 6 in the ETR− group (15%).

### Outcomes based on responses at 6 months

**Fig. 2:**
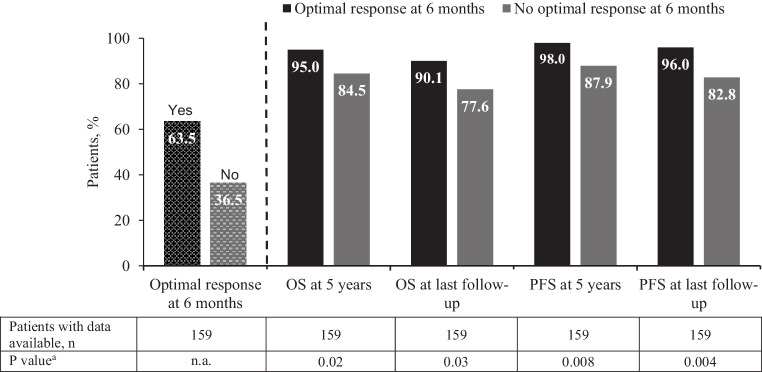
OS and PFS at 5 years and at last visit based on the 6‑month response. *n.a.* not assessed, *OS* overall survival, *PFS* progression-free survival. ^a^For all descriptive statistical comparisons of categorical data (frequencies and percentages) χ^2^-tests were used. Continuous data ware compared using Mann-Whitney U-tests

Of the 159 patients with available data for the 6‑month visit, 101 (63.5%) reached an optimal response at 6 months, whereas 58 (36.5%) did not. Patients in optimal response at 6 months achieved higher OS and PFS rates at 5 years compared with patients who had not reached an optimal response at 6 months (OS: 95% vs. 84.5%, *P* = 0.02; PFS: 98.0% vs. 87.9%, *P* = 0.008). Similar results were seen at the last follow-up with higher OS and PFS rates in patients with optimal response vs. those without optimal response at 6 months (OS: 90.1% vs. 77.6%, *P* = 0.03; PFS: 96.0% vs. 82.8%, *P* = 0.004) (Fig. 2).

### Outcomes based on Sokal risk score

**Fig. 3:**
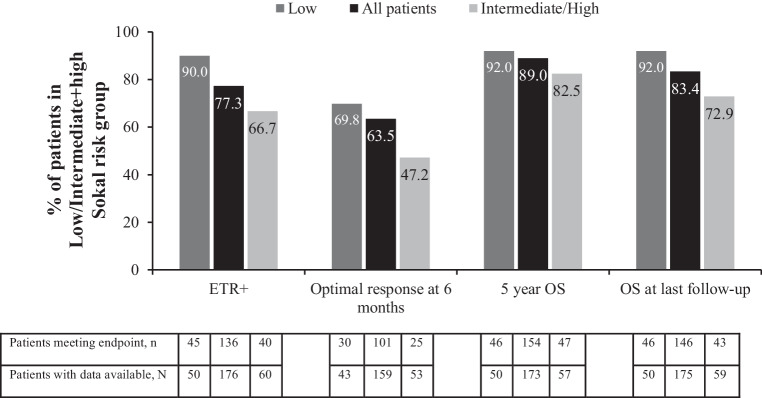
Key outcomes based on Sokal risk scores.^a,b^ *ETR* early treatment response (partial cytogenetic response and/or BCR-ABL1^IS^ ≤ 10% at 3 months), *OS* overall survival. ^a^Percentages were calculated based on number of patients evaluable for each parameter at that particular time point. ^b^Patients in warning and failure groups were combined

Sokal risk scores were available for 110 patients (ETR+ group: *n* = 85; ETR− group: *n* = 25). The majority of patients in the ETR+ group had low and intermediate risk scores (low, 52.9%; intermediate, 34.1%; high, 12.9%), whereas ETR− patients predominantly had intermediate or high risk scores (low, 20.0%; intermediate, 52.0%; high, 28.0%). For further analyses, the intermediate and high risk groups were combined due to the low number of patients in these groups. The majority of patients in the intermediate/high risk group had intermediate risk scores. More patients in the low risk group than in the intermediate/high risk group achieved ETR (low risk group 90% (45/50 patients) vs intermediate/high risk group 66.7% (40/60 patients)) and an optimal response at 6 months (low risk group 69.8% (30/43 patients) vs intermediated/high-risk group 47.2% (25/53 patients)). The rates of OS at 5 years were higher in the low risk group (low-risk group 92.0%, 46/50 patients vs intermediate/high-risk group 82.5%, 47/57 patients), with even higher differences in the OS at the last follow-up (low-risk group 92.0%, 46/50 patients vs intermediate/high-risk group 72.9%, 43/59 patients) (Fig. 3).

### Kinetics of responses

**Fig. 4:**
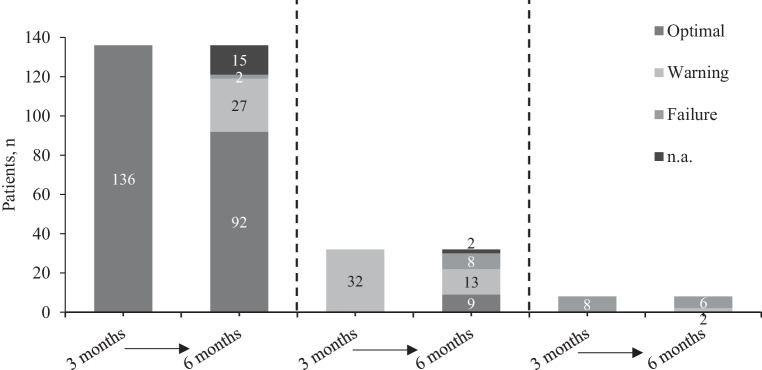
Kinetics of responses between months 3 and 6. *n.a.* not assessed

The ELN has defined molecular and/or cytogenetic criteria for responses (optimal, warning and failure, respectively) to TKI therapy at certain time points [1]. Interestingly, the majority of patients in the warning category at 3 months—defined as BCR-ABL1 >10% and/or 36–95% Ph+ metaphases—(*n* = 32) remained in warning category at 6 months (*n* = 13) or had a treatment failure (>95% Ph+ metaphases; *n* = 8) (Fig. 4). None of the patients in the warning category at 3 months switched to another TKI before month 6, and only 2 patients had a dose escalation of imatinib to 600 mg/day; however, 1 patient remained in the warning category and 1 patient had treatment failure at 6 months.

### Therapy at last documented visit

**Fig. 5:**
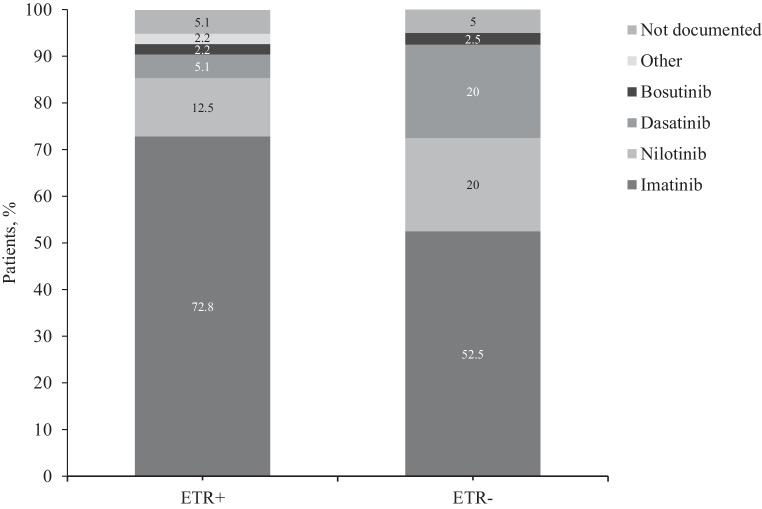
Therapy at last documented visit. *ETR* early treatment response (partial cytogenetic response and/or BCR-ABL1 ≤ 10% at 3 months)

At the last documented visit, more patients had been switched from imatinib to another TKI in the ETR− group than in the ETR+ group. In total, 120 patients were still on imatinib (ETR+ patients, 99, 72.8%; ETR− patients, 21, 52.5%, *P* *=* 0.019, 25 were on nilotinib, ETR+, 17, 12.5%; ETR−, 8, 20.0%), 15 were on dasatinib (ETR+, 7, 5.1%; ETR−, 8, 20.0%), and 4 were on bosutinib (ETR+, 3, 2.3%; ETR−, 1, 2.5%). The TKI status had not been documented for 9 patients, and 6 patients were receiving other drugs (Fig. 5).

### Concordance of BCR-ABL1^raw^ and BCR-ABL1^IS^

An additional analysis was performed to assess the feasibility of using BCR-ABL1^raw^ values for scoring the early responses at 3 and 6 months. Similar percentages of patients were found to have achieved ETR+ when using only BCR-ABL1^raw^ values vs. only BCR-ABL1^IS^ values (raw: 76.8% ETR+, 23.2% ETR−; IS: 71.6% ETR+, 28.4% ETR−). The OS at 5 years was also comparable between patients in whom response to TKI was analyzed by using BCR-ABL1^raw^ and patients in whom BCR-ABL1^IS^ values were obtained (raw: 87.1% for ETR+, 78.9% for ETR−; IS: 95.7% for ETR+, 79.3% for ETR−). This analysis supported the approach of using BCR-ABL1^raw^ values for scoring the early treatment responses in patients for whom IS values or cytogenetic data were unavailable (which affected 33 of 176 patients for ETR).

## Discussion

This study aimed to elucidate whether the survival benefit seen with early responses in controlled clinical trials [2–10] can also be translated into daily clinical practice in Austria. Therefore, this study retrospectively analyzed charts from 12 major institutions across Austria of patients diagnosed with CML-CP between 2004 and 2010. As quantification of BCR-ABL1 levels by IS was not established at all participating sites during the project period, this study evaluated ETR (BCR-ABL1 ≤10% and/or ≤35% Ph+) instead of a purely molecular response at 3 months, which is in concordance with the 2013 ELN criteria [1].

Of note, in this study the substitution of non-IS BCR-ABL1 values (BCR-ABL1^raw^) for scoring early responses of patients for whom neither BCR-ABL1^IS^ nor cytogenetic data were available was accepted. This approach was supported by an analysis in which similar scoring results were obtained with the different BCR-ABL1 measurements. It should be mentioned, however, that this approach was only used for scoring early responses, where slight numerical variations have a smaller impact than at later treatment stages, when BCR-ABL1 values are generally much lower.

Overall, of 176 evaluable patients 136 (77.3%) achieved ETR at 3 months and 40 (22.7%) did not. This was in line with results obtained in large randomized clinical trials (ENESTnd, 67% of patients on imatinib reached EMR [4, 9]; DASISION, 64% on imatinib reached EMR [2, 10]; BFORE, 57.3% on imatinib reached EMR [14]). It is of note that a substantial number of patients on imatinib do not reach optimal early responses in daily routine. Also, the negative impact of intermediate or high risk Sokal scores at diagnosis seen in clinical trials was replicated in this study. Patients in the low-risk group were more likely to reach ETR and have higher 5‑year OS and PFS rates compared with the intermediate/high-risk group.

Patients in the ETR+ group had higher 5‑year OS and PFS rates than patients in the ETR− group (92.5% vs. 77.5%, *P* = 0.018). In the imatinib arm of the ENESTnd trial, the OS and PFS rates at 5 years were >95% for patients reaching EMR, whereas the OS was 79.1% and PFS was 79.3% for patients who failed to reach EMR [4]. Similarly, in the imatinib arm of the DASISION trial, the 5‑year OS and PFS rates of patients reaching EMR were 95.4% and 93.1%, respectively, and were 80.5% and 71.9%, respectively, for patients not reaching EMR [2]. Within the limits of cross-trial comparisons, the measured efficacy parameters obtained in this study appear to be comparable. The 5‑year OS and PFS calculated for the R‑EFECT study are slightly lower than that seen in the large clinical trials. This is possibly driven by the more heterogeneous unselected patient population, especially including those with more comorbidities seen in daily clinical practice. Notably, comorbidities have a strong impact on survival in CML patients treated with TKIs, although this holds true primarily for the second and third generation TKIs [15–17] but not for imatinib [18–20]. Overall, the results confirm the efficacy and long-term treatment outcome of imatinib and show that the predictive value of reaching ETR for better survival does translate to daily clinical routine.

It is not unexpected that patients failing ETR were more likely to switch from imatinib to another TKI. Interestingly, patients who were in the warning category at 3 months as per ELN guidelines [1] were more likely to remain in the warning category or worsen leading to treatment failure by 6 months, rather than to improve to an optimal response. Of the 32 patients in warning at 3 months, 13 were still in warning, 8 had a treatment failure and only 9 showed an optimal response by 6 months. None of the 32 warning patients have switched to another TKI before the 6‑month visit. Interpretations in this respect have to be made with caution since for large parts of the diagnosis period of this study, imatinib was the only approved TKI in Austria. It remains to be determined if a consequent early switch to other TKIs in patients failing ETR is associated with an improved outcome both in clinical trials and in current routine in CML care. Currently, only limited data are available suggesting that the switch to a second generation TKI may improve outcome for patients not achieving an ETR [21].

In total 19 patients (10.8%) died within 5 years, including 4 (2.3%) that were labeled as CML-related deaths (3 in ETR+ group, 2.2%, 1 in the ETR− group, 2.5%). In the imatinib arm of the ENESTnd study, 22 of the 283 patients (~8%) died by 5 years, including 16 (5.7%) CML-related deaths [4]. Similarly, in the imatinib arm of the DASISION study, 26 of 258 patients (10%) died by 5 years and 17 (6.6%) of these deaths were CML-related [2]. Due to the retrospective nature of the current study, attribution of a cause of death from routine records may have been difficult and must therefore be considered with caution.

In summary, these data from a real-life setting support the findings from randomized trials demonstrating that ETR achievement is associated with superior OS and PFS. Patients who achieved ETR are less likely to switch from imatinib to other TKIs, and patients who fail to achieve ETR should be monitored closely and treated according to available guidelines.

### Limitations

Since this study relied exclusively on retrospective data, the observations made here have to be complemented with prospective data before drawing definitive conclusions. Prospective registries might be able to close this gap. In addition, according to the Austrian Pharmaceutical Act, this was an observational study with no hypothesis testing and no control group. There were no local law requirements for monitoring [9]. Consequently, no monitoring was applied and source data verification was not applicable. Missing data were not queried, resulting in a deliberate number of missing data points. A total of 12 major CML treatment centers in Austria participated in the study; however, a bias in the patients selected cannot be excluded.
